# Risk Factors for a Higher Dietary Acid Load (Potential Renal Acid Load) in Free-Living Elderly in Poland

**DOI:** 10.3390/nu16193409

**Published:** 2024-10-08

**Authors:** Katarzyna Rolf, Olga Januszko

**Affiliations:** 1 Department of Food Technology and Human Nutrition, University of Rzeszow, Zelwerowicza 4 Street, 35-601 Rzeszow, Poland; 2 Department of Human Nutrition, Warsaw University of Life Sciences—SGGW, Nowoursynowska 159c Street, 02-787 Warszawa, Poland; olga_januszko@sggw.edu.pl

**Keywords:** potential renal acid load, PRAL, elderly, dietary acid load, frailty syndrome

## Abstract

Background: Dietary composition is one of the factors influencing the acid–base balance of the body by providing acid or base precursors. One of the methods for assessing the acid-forming potential of a diet is to calculate its potential renal acid load (PRAL). The aim of this study was to identify the sociodemographic, lifestyle, and health factors related to the PRAL. Methods: Dietary intake was assessed among 133 individuals aged 70+ years using the three-day record method. Results: The average PRAL value was 15.7 mEq/day (range from −42.4 to +101.7). The diets of a majority of the participants (71.4%) had acid-forming potential (PRAL > 0). From a univariate analysis, the acid-forming potential of the diets was linked mainly to women (65.3% in PRAL > 0 group vs. 10.5% in PRAL < 0 group), people using dietary supplements, those who consumed alcohol, those who assessed their health as being at least good, people with osteoporosis, those hospitalized during the previous year, and those with rather lower physical activity. Conclusions: From a multivariate analysis, gender was the strongest predictor of an acid-forming diet, but the following also contributed: an average self-rated health status (compared to good), a good health status (compared to poor), alcohol drinking, hospitalization, lack of nutritional knowledge, and, to a lesser extent, non-frail status (compared to pre-frail). Therefore, more extensive nutritional education in the identified groups is required.

## 1. Introduction

The relationship between one’s diet and healthy aging is becoming increasingly clear from recent studies. The acid–base balance in the human body has been found to be very important for maintaining health. Diet composition has long been known to influence the acid–base balance by providing acid or base precursors. Scientists say that we must maintain a constant blood pH, i.e., between 7.35 and 7.45, for the processes taking place in our body to proceed properly. The ability of food to acidify or alkalize the body can be measured by the potential renal acid load (PRAL) index, which determines the acid loads of foods or diets. A decrease in the PRAL in the negative (−) direction indicates that the diet is shifting to alkaline values, while its increase in the positive (+) direction indicates that the diet is shifting to acidic ones [1]. Another indicator used to estimate the dietary acid load of a diet is the net endogenous acid production (NEAP), which predicts the absolute rate of metabolic acidosis [2].

A growing number of studies on various populations around the world have indicated a correlation between the dietary acid load and health conditions. For example, it is known that bone mineral density (BMD) is closely related to diet composition, as are the PRAL and NEAP indexes [3,4,5]. It has been reported that adherence to a Mediterranean diet, characterized as being more alkaline, is associated with better bone health [6,7,8].

However, a Western diet is characterized by a high consumption of highly processed products simultaneously with a low content of fruits and vegetables [9,10]. Because the body’s acid load is greater with such a diet, it can lead to chronic metabolic acidosis, which, in turn, is associated with the development of insulin resistance, diabetes, hypertension, chronic kidney disease, bone disease, low muscle mass, and other complications [11].

Several meta-analysis studies have investigated the association between PRAL and some CVD risk factors. A high PRAL and NEAP can be considered independent risk factors for high blood pressure and hypertriglyceridemia. The association between the dietary acid load and cardiometabolic risk factors is age- and gender-dependent [12,13]. However, not all studies are consistent in this respect. In a study of the adult Polish population, no relationship was found between the dietary acid load content and the occurrence of cardiovascular diseases and their risk factors [14]. Metabolic acidosis seems to foster diabetes [14,15,16] and cancer [17,18,19]. However, it should be remembered that a diet with high PRAL values (very alkalizing) is also negative for one’s health; for example, it can significantly increase the risk of death due to CVD [20].

This review of the available scientific studies assessing the relationship between PRAL or NEAP and the occurrence of chronic diseases and metabolic disorders seems extensive, but the results are not always unambiguous. However, there is a lack of research on the impact of some other factors, like nutritional knowledge, marital status, and lifestyle, on the PRAL index, especially among older people. Therefore, the aim of our study was to check whether, in a selected group of elderly adults, the PRAL categories are influenced by sociodemographic, lifestyle (including diet), and health factors.

## 2. Materials and Methods

### 2.1. Participants

Recruitment was carried out in 2020–2021. Participants were recruited through information provided at seniors’ clubs and Universities of the Third Age, as well as advertisements in the local press. All the subjects taking part in this study were volunteers. All the participants signed an informed consent form prior to their inclusion in this study. This study’s participants were free-living, responsible for their own dietary choices, over 70 years of age, and free of diseases compromising 2-year survival. The exclusion criteria were overt diseases such as cancer or dementia, a history of severe heart disease, organ failure (unstable, renal, respiratory, liver), diabetes mellitus type I, insulin-dependent diabetes mellitus, the chronic use of corticosteroids or the recent use of antibiotics, a recent change in habitual medication use, the presence of a food allergy or intolerance necessitating a special diet, and not being competent for making their own decisions. We used screening questionnaires to verify that none of the exclusion criteria were present.

A total of 163 participants volunteered for this study; however, 26 participants were found to not meet the inclusion criteria, and 4 dropped out during this study. Ultimately, 133 respondents were included in this study of apparently healthy individuals aged 70–97 years.

The Ethical Committee of the Warsaw University of Life Sciences approved this in 2019 (Resolution No. 50p/2019). This study was carried out in accordance with The Code of Ethics of the World Medical Association (Declaration of Helsinki).

### 2.2. Data Collection

The data were collected using a questionnaire that included 35 questions and consisted of the following sections: (1) sociodemographic characteristics, (2) health and lifestyle status, (3) self-reported level of nutritional knowledge and eating habits, and (4) dietary supplement (DS) usage.

(1) The sociodemographic questions regarded gender, data on age, spousal status, education level, and place of living.
(2) The health and lifestyle questions provided data on self-reported health status, occurrence of chronic diseases, hospitalization within the previous year, cigarette smoking, alcohol drinking, self-reported physical activity levels, weight, and height. To help respondents choose the appropriate physical activity category, each category gave examples of types of exercise and the number of hours spent weekly on these activities. The body mass index (BMI) was calculated according to a specific formula based on height (m) and weight (kg) and interpreted according to the World Health Organization classification [21].
(3) The simplified Nutritional Appetite Questionnaire (SNAQ) was used to identify nutritional risk, developed as a self-assessment screening tool which is easy to administer without laboratory measurements [22].
(4) Participants were asked about all DSs taken six months before the study. Information was collected on the name and brand, the form used (i.e., capsules, tablets, powder, etc.), the duration of use, and the reason for usage.

### 2.3. Dietary Assessment

The three-day food record method was used to collect data about food consumption, covering two non-consecutive weekdays and one weekend day. In contrast to Food Frequency Questionnaires and 24-h recalls, food records minimized reliance on memory, as food intake was recorded by the elderly at the time when the foods were eaten [23].

To encourage participation in the food consumption records, free nutritional advice was offered to those who completed them.

The respondents were instructed on how to provide detailed information about the food and drinks consumed and determine the portion sizes (using kitchen scales or household measures). The participants were asked to return their food record questionnaires within one month of the recruitment meeting.

Researchers verified the returned questionnaires. Imprecise or missing information on portion size was corrected together with the respondent using a photo album of products and dishes [24].

An analysis of data from the food record questionnaires was carried out based on “Polish tables of composition and nutritional value” [25].

### 2.4. Dietary Acid Load

Using the potential renal acid load (PRAL) of a diet, renal net acid excretion (NAE) can be estimated, without laboratory testing. The formula used to calculate the PRAL scores was as follows [26]:

PRAL (mEq/d) = 0.4888 × protein intake (g/d) + 0.0366 × phosphorus (mg/d) − 0.0205 × potassium (mg/d) − 0.0125 × calcium (mg/d) − 0.0263 × magnesium (mg/d)

Based on previous works in the literature [26], participants were separately divided into two groups based on the classification of PRAL value on PRAL < 0 (base-forming potential) and PRAL > 0 (acid-forming potential).

In addition, we also estimated the NEAP value, which is the second model generally used to calculate the dietary acid load of foods; the formula is shown below [2]:

$$NEAP\left(mEq/d\right)=\frac{54.5\times protein\left(g/d\right)}{potassium\left(mEq/d\right)}-10.2$$

### 2.5. Frailty Syndrome

Frailty syndrome (FS) was evaluated according to the five criteria proposed by Fried et al. [27]: (1) unintentional weight loss (shrinking); (2) weakness; (3) exhaustion; (4) low walking speed; and (5) physical inactivity. Individuals were considered to be “frail” if they met ≥3 criteria, and those who had 1–2 criteria were classified as “pre-frail”. If the participants did not have any of the described characteristics, they were categorized as “non-frail”.

### 2.6. Statistical Analysis

The data were analyzed using Statistica software version 13.3 (TIBCO Software Inc., Palo Alto, CA, USA). The results were considered statistically significant at *p* ≤ 0.05, while for 0.1 > *p* > 0.05 the results were considered as a trend [28].

To measure statistical significance between groups of PRAL and different factors, parametric and non-parametric tests were used, as appropriate (Mann–Whitney U test for quantitative variables and Pearson’s chi-squared test for qualitative variables). The mean and standard deviation (SD) were shown for continuous data and the percentage for categorical variables.

Associations between potential risk factors and PRAL score were examined using logistic regression with data presented as OR and 95% CI. The probability factor for the occurrence of the event was an acid-forming diet (PRAL > 0). The final multivariate model included factors with univariate *p*-values ≤ 0.05 and, additionally, nutritional knowledge and all diseases as factors that can be related to dietary habits. Additionally, multiple linear regression analyses were performed using the same factors as in the multiple logistic regression models. Analyses were performed separately for PRAL and NEAP values.

## 3. Results

### 3.1. The Characteristic of PRAL Groups

This study was conducted among 133 people aged >70 years, including 66 women (49.6%). The average PRAL value in the entire group was 15.7 mEq/day (range from −42.4 to +101.7). The average NEAP value was 40.9 mEq/day (5.9–109.6), and it was higher among men. The average value of PRAL among women was 28.9 and men 2.6 (Table 1). The diets of a majority of participants (71.4%) had an acid-forming potential (PRAL > 0). The PRAL groups differed significantly with regard to gender (*p* < 0.000) but not age, educational level, residential area, household size, and spousal status (*p* > 0.05) (Table 2).

Significantly more participants in the PRAL > 0 group (almost 79%) compared to PRAL < 0 (just under 58%) used dietary supplements. The potential of an acid-forming diet was also significantly correlated with alcohol drinking. There was no difference between the PRAL index and nutritional knowledge, SNAQ result, and smoking. A slight relationship was observed between the PRAL index and the self-assessment of physical activity (*p* = 0.0989). Respondents in the PRAL < 0 group tended to be more active (Table 3).

Among the health factors, the PRAL score was only associated with health status, hospitalization, and osteoporosis. In the PRAL > 0 group, compared to the PRAL < 0 group, there were significantly more elderly people with osteoporosis (26.3% vs. 10.5%) and less who had been hospitalized the previous year (13.7% vs. 28.9%). Moreover, a larger percentage of people in the PRAL > 0 group rated their health as average (44.2) and, in the PRAL < 0 group, as good (52.6). Additionally, there were no subjects in the PRAL > 0 group (Table 4).

Among the analyzed nutrients, people with PRAL > 0, compared to those with PRAL < 0, were characterized by a significantly lower intake of water (1853.1 vs. 2377.4 mL/d), sodium (2399.7 vs. 3168.1 mg/d), and vitamin B6 (2.1 vs. 3.5 mg/d) and higher % energy from fat (34.6 vs. 30.9). The higher PUFA intake in the PRAL > 0 group was borderline statistically significant (*p* = 0.0587) (Table 5).

### 3.2. The Relationship between Covariates and the PRAL Score

The multivariate odds ratios (ORs) of the acid-forming potential of a diet, by logistic regression, in a study group of elderly are presented in Table 6. The strongest association was found between the PRAL index and gender (*p* < 0.000). Compared to men, the diet of women had a higher PRAL index, which means over 13 times more acid-forming potential in their diet (OR: 13.50; 95% CI: 3.03–41.73). Respondents self-assessing their health status as being average were more than 10 times more likely to have an acidifying diet, while elderly with poor health status more often had an alkaline diet (OR 10.64, 95% CI 1.74–55.34 and OR 0.12, 95% CI 0.05–0.80, respectively). Compared to robust elderly participants, the pre-frail status of respondents was slightly more correlated with the alkaline-forming potential of a diet (*p* = 0.055; OR: 0.54; and 95% CI: 0.12–1.42). The prevalence of an acid-forming potential of a diet was significantly lower among people with osteoarthritis (*p* = 0.016; OR: 0.36; and 95% CI: 0.16–0.83) and a little lower among people with thyroid diseases (*p* = 0.058; OR: 0.31; and 95% CI: 0.09–0.99) and hypertension (*p* = 0.093; OR: 0.52; and 95% CI: 0.24–1.12). Hospitalization was significantly associated with an increased risk of an acidifying diet (*p* = 0.035; OR: 2.88; and 95% CI: 1.08–7.70). Alcohol drinking increased the chance of an acidifying diet by about 50% (OR: 0.42; 95% CI: 0.19–0.94). The acid-forming potential of diet tended to be 6.81-fold (95% CI: 1.51–13.67) higher among those who declared a lack of nutritional knowledge vs. good knowledge (*p* = 0.012). However, respondents with average knowledge tended to more often have an acid-forming potential in their diet of about 80% (*p* = 0.005; OR: 0.16; and 95% CI: 0.05–0.58). There was also a strong relationship between the NEAP value and the PRAL groups (*p* = 0.001; OR: 1.14; and 95% CI: 1.05–1.23).

When PRAL was used as a continuous variable, similar correlations were found. However, for the NEAP value, some results were different. As the PRAL value increased, the NEAP value increased significantly and vice versa. Compared to men, the diet of women had a much higher PRAL value (*p* < 0.000, beta = 0.548) but a lower NEAP value (*p* < 0.000, beta = 0.393). Self-rated health status had a significant effect on the PRAL but not on the NEAP. For frailty syndrome, the opposite was true. Please refer to Table S1 for more information.

## 4. Discussion

The gender of our respondents was the most important factor influencing the value of the PRAL index, i.e., the acid or alkaline potential of a diet, but also the NEAP value. A female gender increased the PRAL value, making the chance of having an acid-forming diet, on average, 13 times higher and, when extrapolating the results to the general population, up to 41 times higher. This is of particular concern given the high incidence of osteoporosis and the associated risk of hip fractures in women [29]. Our univariate analysis showed an association between osteoporosis and PRAL; osteoporosis was more prevalent among people with an acid-forming diet. Simultaneously, women were more likely to have an acidifying diet. Although there was no relationship between PRAL and osteoporosis in our regression analysis, this relationship is logical, because gender was a confounding variable, meaning that we can confirm the association of the PRAL value with gender.

The impact of one’s diet on bone health is the subject of much debate. A diet with an acid-forming potential may significantly increase the risk of bone loss [3]. But, not all studies support the acid–base hypothesis of bone loss [30,31]. A systematic review of the study on the association of PRAL with bone health, including hip fractures, which are often associated with osteoporosis in older adults, found no statistical significance [32]. Simultaneously, there are some studies confirming such a relationship [4,33]. It has been suggested that older people, who are at greater risk of presenting declining kidney function, lower muscle mass, and osteoporosis, may potentially benefit from an acid load-limiting diet [34]. We obtained inconclusive data regarding frailty syndrome. In the logistic regression analysis, but not in the linear analysis, non-frail elderly participants had a slightly higher chance of having a diet with PRAL > 0 than pre-frail individuals. However, when analyzing the NEAP value, we obtained an inverse correlation—pre-frail people tended to have lower NEAP values, while frail people tended to have higher ones. Usually, frailty syndrome is associated with a higher dietary acid load in contexts such as, for example, Japan [35] or China [3]. It should be remembered that an acid-forming diet inhibits the synthesis of muscle proteins and increases their degradation [36], which may contribute to the development of frailty syndrome in the future or intensify its symptoms.

The average NEAP of the study population was 40.9 mEq/day, which was slightly lower than in a typical Western diet (50 mEq/day) [2] but similar to another study [30,37]. Additionally, the calculation of NEAP and PRAL uses protein intake as a surrogate of sulfur amino acid production, and we know that a higher protein intake is recommended in older age [38].

The lower value of PRAL among men may indicate that our male respondents’ intake included less protein (mainly meat and dairy products) and/or more vegetables and fruits than women, so their diet was better. In our entire study group, people with PRAL > 0 had higher NEAP values, and the directly proportional relationship is also confirmed by other studies [30,35]. However, our male respondents had higher NEAP values, which, in turn, may suggest a higher protein intake than women. Most studies show that women tend to have a better diet [39,40]. However, it should be highlighted that the diet of seniors, regardless of gender, has been inconsistent in terms of official recommendations for many years, both in Poland [41,42,43] and around the world [22,44]. Even though meat consumption decreases with age, older men still consume more meat than women [9]. With regard to the acid–base balance, the cited research shows that seniors primarily do not eat enough vegetables and fruits.

The results of our more detailed analysis of consumption are disturbing. Despite the lack of association between PRAL and the intake of most nutrients, we found an abnormal ratio of SFA:MUFA:PUFA fatty acid ratio; indeed, in the whole population, it was 1:1.09:0.50. However, the ideal ratio should be 1:1.5:1 [45], so our respondents did not eat enough PUFA and MUFA. Such abnormalities have also been found among Swedish men [30]. Vitamin D intake was also well below the recommended 15 ųg/d [46]. This is confirmed by other studies in Poland [41] and other countries [30,37]. However, the lack of statistically significant differences for most minerals was probably due to a high standard deviation.

We were surprised that people who rated their health as being poor, compared to good, mostly had an alkalizing diet. But, people reporting average health, compared to good health, had an acidifying diet. The same results were found in the linear regression analysis for the PRAL value. The explanation may be that poor health is more likely to force us to take better care of ourselves or that, among older adults with poor health and unhealthy dietary habits, women make up the majority [47]. This is again evidence of the strong influence of gender on nutrition and, consequently, on the PRAL index. Other studies on the relationship between the value of PRAL and gender are inconclusive. There was no statistically significant difference in studies conducted in the Netherlands among people ≥55 years of age [48] and in Germany among adults aged 18–79 years [49]. In contrast, women had a lower mean PRAL value (more alkaline) in the UK in the 42–82 year age group [50] or in China among those aged 52–83 years [3]. The discrepancies may result from the age groups, the health status of the respondents, or their income, but also from external conditions, such as the season. All of these may be modulators of the diet. However, in another study conducted among community-dwelling people in Sweden, aged >70 y, like our study, men had higher PRAL and NEAP values [30]. In the study by Shea et al. [51], women had significantly lower urinary NAE values, indicating lower acidification of the body, than men. Men usually intake more meat products [52], but perhaps the women in our study consumed extremely low amounts of fruits, the intake of which is correlated with an acid–base balance [51]. This may be explained by men attending day care senior centers more often. In such places, individuals receive regular meals, including vegetables and fruits. Perhaps, older people who do not go to senior centers avoid this food group due to factors such as economic reasons, among others. However, despite the overall better quality of women’s diets, some studies indicate that men consume fruit and vegetables more frequently [41].

It is worth noting that respondents assessing their health as being average, compared to good, more often had an acid-forming diet, but people with poor health (compared to good) had an alkaline-forming diet. This is partially confirmed by the study by Zhao and Andreyeva [53], as, among older Americans, health deteriorated as diet quality decreased. However, Jeruszka-Bielak et al. [40] found no relationship between the self-assessment of health and the quality of a diet among Polish seniors—people eating many or few vegetables and fruits rated their health similarly. Because our study was a cross-sectional study, it is not known whether the acid-producing diet was a cause or a consequence of poor health. However, it is known that continuing such a dietary pattern may further worsen health. Interestingly, women more often assess their health as being poor, compared to men [10]. Moreover, a poorer health is often associated with a worse financial situation, which influences food choices, especially among older people [54,55]. The low income of most seniors results in the choice of cheaper and often highly processed foods [55], which are, among other characteristics, poor in minerals, meaning that they have a high potential to acidify the body [56]. Despite possessing quite good nutritional knowledge, over 70% of seniors in our study had a diet that promoted acidification. While many older consumers make poor financial choices which may be partly explained by cognitive impairment [57], our study was conducted among people living independently, actively, e.g., attending senior clubs or Universities of the Third Age, so they were unlikely to have any cognitive disorders.

Although we did not demonstrate a statistically significant relationship between spousal status and PRAL, a slightly higher percentage of people living in a relationship had a healthier diet, with alkaline-forming potential. Other studies confirm the positive influence of a spouse on the quality of one’s diet [58,59]. A decline in fruit and vegetable intake among elderly people without a spouse is usually more apparent in terms of the variety rather than quantity consumed [9], so it should not impact to PRAL index.

The inverse relationship between self-assessed “good” and “average” nutritional knowledge and the PRAL index was surprising to us. Respondents with high levels of knowledge should have had a better diet, but they had a greater tendency to present an acid-forming diet than the respondents who had “average” knowledge. A higher self-rated knowledge may be associated with a higher economic status, which, according to some studies, is associated with eating more meat [60]. However, other studies show that a higher socioeconomic status is associated with greater fruit and vegetable intake [54,61]. In other studies, 80% of Poles have been reported to believe to eat properly, and about 70% of people have rated their health as being at least good. Simultaneously, insufficient knowledge of the risks caused by an improper diet and low physical activity has been demonstrated, indicating that lifestyle knowledge does not correlate with its practice [62,63]. Perhaps, our respondents’ high self-rating was only a result of many of them attending a University of the Third Age. What is also surprising is that we found no relationship between nutritional knowledge and PRAL and NEAP as continuous variables.

Dietary habits learned from one’s family home and formed during adolescence determine people’s lifestyle and diet in adulthood. These habits are difficult to change; therefore, educational activities should be carried out in younger age groups [64].

Although our study provides ambiguous results for some indicators, it should be noted that kidney function is reduced with age, so even a slightly alkaline diet, as measured by the PRAL or NEAP indices, may lead to the acidification of the organism in older people [65].

The composition of one’s diet influences the net endogenous acid production. One of the limitations of our study is its cross-sectional nature, which makes it impossible to directly assess the urinary net acid excretion (NAE). But, the PRAL value is strongly related to the NAE, which makes our study reliable. Another weakness of this study may be its small group of respondents, because it was conducted only among 133 elderly people. One of the reasons for the aversion of older people to participating in research, especially in Poland, is the lack of willingness to be “the object of experiments” [66]. Another reason may be the recruitment method solely through volunteers. However, such a selection method can also be an advantage, because such people are more willing to take part in this type of research and fill in the questionnaires more meticulously, which translates into a greater credibility of the results. In addition, our study included healthy individuals over 70 years in age. Elderly people have many disabilities that make it difficult for them to participate in such studies. Of course, our results may reflect different dietary habits or a selection bias for more health-oriented individuals who are willing to participate in our survey. However, an undoubtable advantage of our study is the equal participation of women and men, despite the overall small size of the group. This is not insignificant, as there are more women than men in Poland’s population [67]. Another strength of our study is the three-day record method used to assess daily intake, including non-consecutive days, which allowed us to take into account a greater diversity in consumption and made the obtained data more accurate.

## 5. Conclusions

The diet of most respondents had an acid-forming potential (PRAL > 0), which was not surprising, as the Western diet model is the dominating one in Poland. The average PRAL value for the entire study population was about 16 mEq/day.

Taking into account various potentially confounding variables, gender was the strongest predictor of an acid-forming diet. Surprisingly, significantly more women than men had an acidifying diet. Among women, the average PRAL value was almost 30 mEq/day, and among men, it was less than 3 mEq/day.

Logistic regression showed an increased risk of having a diet with PRAL > 0 in the following cases: people with an average self-rated health status (compared to good) or a non-frail status (vs. pre-frail), the presence of any thyroid disease, osteoarthritis, alcohol drinking, and the lack of nutritional knowledge. However, an average level of nutritional knowledge (compared to good) lowered the risk of having an acid-forming diet. Linear regression confirmed some, but not all, of these results.

Due to the correlation between the acid-forming effect of a diet and various factors (sociodemographic, lifestyle, and health), initiatives to improve people’s diets are needed. Therefore, there is a need for more extensive nutritional education in the identified groups, especially aimed at increasing the consumption of vegetables and fruits, which are alkaline-forming products. It also seems that it would be a good idea to have routine laboratory tests carried out among older people to more accurately determine the acid–base balance of the body.

## Figures and Tables

**Table 1:** PRAL and NEAP values by gender.

Factor	Total *n*= 133	Women *n* = 66 (49.6%)	Men *n* = 67 (50.4%)
PRAL			
Average (SD)	15.7 (26.0)	28.9 (23.2)	2.6 (21.7)
Range	−42.4–101.7	−28.3–101.7	−42.4–89.0
NEAP			
Average (SD)	40.9 (14.9)	39.1 (16.1)	42.6 (13.6)
Range	5.9–109.6	5.9–109.6	23.2–78.9

PRAL—potential renal acid load [mEq/day]; NEAP—net endogenous acid production [mEq/day]; and SD—standard deviation.

**Table 2:** Sociodemographic characteristics by PRAL index of diet.

Factor	Total *n* = 133	PRAL > 0 Acid-Forming Potential *n* = 95 (71.4%)	PRAL < 0 Base-Forming Potential *n* = 38 (28.6%)	*p*-Value
Gender				<0.0000 ^1^
Women	66 (49.6)	62 (65.3)	4 (10.5)	
Men	67 (50.4)	33 (34.7)	34 (89.5)	
Age [years]				0.9161 ^2^
Average (SD)	74.5 (4.9)	74.7 (5.3)	74.1 (3.6)	
Range	70–97	70–97	70–89	
Education level				0.9295 ^1^
Primary	11 (8.3)	8 (8.4)	3 (7.9)	
Secondary	45 (33.8)	33 (34.7)	12 (31.6)	
Higher	77 (57.9)	54 (56.9)	23 (60.5)	
Residential area				0.8013 ^1^
City >100,000 inh.	111 (83.4)	78 (82.1)	33 (86.8)	
City <100,000 inh.	9 (6.8)	7 (7.4)	2 (5.3)	
Village	13 (9.8)	10 (10.5)	3 (7.9)	
Household size [number of members]				0.9009 ^2^
Average (SD)	2.01 (1.14)	2.03 (1.17)	1.95 (1.09)	
Range	1–7	1–7	1–7	
Spousal status				0.1199 ^1^
Without spouse	56 (42.1)	44 (46.3)	12 (31.6)	
With spouse	77 (57.9)	51 (53.7)	26 (68.4)	

PRAL, potential renal acid load; SD, standard deviation; ^1^, Pearson’s chi-squared test; ^2^, Mann–Whitney U test; and *p*-value ≤ 0.05.

**Table 3:** Variables of lifestyle by PRAL index of diet.

Factor	Total *n* = 133	PRAL > 0 Acid-Forming Potential *n* = 95 (71.4%)	PRAL < 0 Base-Forming Potential *n* = 38 (28.6%)	*p*-Value ^1^
Self-rated physical activity				0.0989
High	23 (17.3)	13 (13.7)	10 (26.3)	
Average	63 (47.4)	44 (46.3)	19 (50.0)	
Low	47 (35.3)	38 (40.0)	9 (23.7)	
Nutritional knowledge				0.5924
Good	26 (19.5)	19 (20.0)	7 (18.4)	
Average	83 (62.4)	57 (60.0)	26 (68.4)	
Lack	24 (18.1)	19 (20.0)	5 (13.2)	
SNAQ				0.1828
Risk of malnutrition	19 (14.3)	16 (16.8)	3 (7.9)	
Not at risk of malnutrition	114 (85.7)	79 (83.2)	35 (92.1)	
Dietary supplement use				0.0135
Yes	97 (72.9)	75 (78.9)	22 (57.9)	
No	36 (27.1)	20 (21.1)	16 (42.1)	
Current smoking				0.8624
Yes	15 (11.3)	11 (11.6)	4 (10.4)	
No	118 (88.7)	84 (88.4)	34 (89.6)	
Alcohol drinking				0.0352
Yes	112 (84.2)	84 (88.4)	28 (73.7)	
No	21 (15.8)	11 (11.6)	10 (26.3)	

PRAL, potential renal acid load; ^1^, Pearson’s chi-squared test; and *p*-value ≤ 0.05.

**Table 4:** Health variables by PRAL index of diet.

Factor	Total *n* = 133	PRAL > 0 Acid-Forming Potential *n* = 95 (71.4%)	PRAL < 0 Base-Forming Potential *n* = 38 (28.6%)	*p*-Value
BMI [kg/m^2^]				0.6291 ^2^
average (SD)	26.6 (4.9)	26.5 (5.2)	26.7 (4.0)	
range	17.6–42.4	17.6–42.4	18.2–35.8	
Weight change				0.3425 ^1^
Yes	79 (59.4)	54 (56.8)	25 (65.8)	
No	54 (40.6)	41 (43.2)	13 (34.2)	
Self-rated health status				0.0014 ^1^
Good	73 (54.9)	53 (55.8)	20 (52.6)	
Average	55 (41.3)	42 (44.2)	13 (34.2)	
Poor	5 (3.8)	0	5 (13.2)	
Diabetes				0.6623 ^1^
Yes	9 (6.8)	7 (7.4)	2 (5.3)	
No	124 (93.2)	88 (92.6)	36 (94.7)	
Hypertension				0.2900 ^1^
Yes	78 (58.6)	53 (55.8)	25 (65.8)	
No	55 (41.4)	42 (44.2)	13 (34.2)	
Thyroid diseases				0.6222 ^1^
Yes	17 (12.8)	13 (13.7)	4 (10.5)	
No	116 (87.2)	82 (86.3)	34 (89.5)	
Osteoporosis				0.0463 ^1^
Yes	29 (21.8)	25 (26.3)	4 (10.5)	
No	104 (78.2)	70 (73.7)	34 (89.5)	
Osteoarthritis				0.1125 ^1^
Yes	33 (24.8)	20 (21.1)	13 (34.2)	
No	100 (75.2)	75 (78.9)	25 (65.8)	
Frailty syndrome				0.1614 ^1^
Frail	13 (9.8)	12 (12.6)	1 (2.6)	
Pre-frail	46 (34.6)	30 (31.6)	16 (42.1)	
Non-frail	74 (55.6)	53 (55.8)	21 (55.3)	
Hospitalization				0.0387 ^1^
Yes	24 (18.1)	13 (13.7)	11 (28.9)	
No	109 (81.9)	82 (86.3)	27 (71.1)	

PRAL, potential renal acid load; SD, standard deviation; ^1^, Pearson’s chi-squared test; ^2^, Mann–Whitney U test; and *p*-value ≤ 0.05.

**Table 5:** Dietary intake of elderly by PRAL index of diet ^1^.

Factor	Total (*n* = 133)	PRAL > 0 Acid-Forming Potential *n* = 95 (71.4%)	PRAL < 0 Base-Forming Potential *n* = 38 (28.6%)	*p*-Value ^2^
PRAL (mEq/d)	15.7 ± 26.0	27.5 ± 20.6	−13.8 ± 8.9	---
NEAP (mEq/d)	40.85 ± 14.9	42.3 ± 15.2	37.1 ± 13.9	0.0835
Nutrient intake				
Energy (kcal/d)	1794.0 ± 659.7	1800.0 ± 624.2	1779.0 ± 750.1	0.4565
Water (ml/d)	2002.9 ± 732.4	1853.1 ± 680.0	2377.4 ± 732.9	<0.0000
Carbohydrates (% of energy)	49.2 ± 7.7	48.6 ± 7.1	50.9 ± 8.9	0.2854
Protein (% of energy)	16.9 ± 3.8	17.0 ± 3.8	16.5 ± 3.7	0.7480
Fat (% of energy)	33.5 ± 7.1	34.6 ± 7.0	30.9 ± 6.7	0.0258
Carbohydrates (g/d)	232.5 ± 94.9	227.7 ± 82.6	244.4 ± 120.8	0.9742
Fiber (g/d)	23.0 ± 10.7	23.6 ± 10.6	21.5 ± 11.1	0.0967
Protein (g/d)	74.1 ± 26.5	75.3 ± 26.9	71.1 ± 25.6	0.4998
Animal protein (g/d)	48.2 ± 20.5	48.7 ± 21.5	46.9 ± 18.1	0.9742
Plant protein (g/d)	25.9 ± 11.2	26.5 ± 11.5	24.2 ± 10.4	0.2448
Fat (g/d)	68.6 ± 30.9	71.3 ± 32.1	61.6 ± 26.7	0.1263
SFA (g/d)	24.3 ± 12.0	24.9 ± 12.5	22.7 ± 10.7	0.3132
MUFA (g/d)	26.7 ± 12.9	27.8 ± 13.3	24.0 ± 11.3	0.1662
PUFA (g/d)	12.2 ± 8.0	13.1 ± 8.5	10.1 ± 6.1	0.0587
Phosphorus (mg/d)	1282.2 ± 478.7	1308.7 ± 478.3	1215.9 ± 479.7	0.2922
Potassium (mg/d)	3365.2 ± 1230.2	3317.6 ± 1144.3	3484.2 ± 1432.7	0.5484
Calcium (mg/d)	726.7 ± 349.7	730.4 ± 358.2	717.3 ± 331.8	0.9781
Magnesium (mg/d)	370.6 ± 175.2	373.1 ± 184.1	364.2 ± 152.8	0.8246
Sodium (mg/d)	2619.2 ± 1462.0	2399.7 ± 1403.6	3168.1 ± 1478.9	0.0018
B6 (mg/d)	2.5 ± 5.1	2.1 ± 1.4	3.5 ± 3.7	0.0207
B12 (µg/d)	4.3 ± 2.3	4.2 ± 2.1	4.5 ± 2.7	0.8233
Folacin (µg/d)	319.4 ± 130.9	317.3 ± 121.8	324.6 ± 153.2	0.7608
Vit. D (µg/d)	5.4 ± 5.8	5.1 ± 5.5	6.3 ± 6.5	0.3704

PRAL, potential renal acid load; NEAP, net endogenous acid production; SFA, saturated fatty acid; MUFA, monounsaturated fatty acid; PUFA, polyunsaturated fatty acid; ^1^ values are means ± SD (standard deviation); ^2^ Mann–Whitney U test; and *p*-value ≤ 0.05.

**Table 6:** Logistic regression results depicting the relationship between covariates and PRAL > 0.

Variable	*p*-Value	OR	95% CI
NEAP	0.001	1.135	1.051–1.227
Gender			
Reference—Men	<0.000	13.504	3.034–41.734
Self-rated health status			
Reference—good			
Average	0.007	10.644	1.738–55.335
Poor	0.003	0.124	0.052–0.801
Frailty Syndrome			
Reference—non-frail			
Pre-frail	0.055	0.536	0.115–1.416
Frail	0.565	1.679	0.211–13.370
Hypertension			
Reference—No	0.093	0.520	0.242–1.115
Diabetes			
Reference—No	0.348	2.093	0.448–8.777
Thyroid diseases			
Reference—No	0.058	0.307	0.095–0.990
Osteoporosis			
Reference—No	0.557	0.734	0.261–2.062
Osteoarthritis			
Reference—No	0.016	0.363	0.160–0.827
Hospitalization			
Reference—Yes	0.035	2.879	1.077–7.697
Alcohol drinking			
Reference—Yes	0.035	0.421	0.188–0.941
Nutritional knowledge			
Reference—good			
Average	0.005	0.165	0.047–0.579
Lack	0.012	6.809	1.511–13.671
Dietary supplement use			
Reference—Yes	0.359	1.415	0.674–2.971

PRAL—potential renal acid load; NEAP—net endogenous acid production [mEq/day]; OR—odds ratio; and CI—confidence interval.

## Data Availability

The original contributions presented in the study are included in the article and Supplementary Materials, further inquiries can be directed to the corresponding author.

## Supplementary Materials

The following supporting information can be downloaded at https://www.mdpi.com/article/10.3390/nu16193409/s1: Table S1: Multiple linear regression results depicting the relationship between the covariates, PRAL, and NEAP.
