# Supplementary material for: Risk Factors for a Higher Dietary Acid Load (Potential Renal Acid Load) in Free-Living Elderly in Poland
Source: Nutrients. 2024 Oct 8;16(19):3409. doi: 10.3390/nu16193409 (PMC11478483; doi:10.3390/nu16193409)
Supplement: Supplementary file 1 [file nutrients-16-03409-s001.zip › nutrients-3194376-supplementary.pdf]

Table S1. Multiple linear regression results depicting the relationship between covariates and PRAL, NEAP.

	PRAL		NEAP	
**Variable**	**p-value**	**beta (95% CI)**	**p-value**	**beta (95% CI)**
**NEAP**	<0.000	0.504 (0.366;0.641)	-	-
**PRAL**	-	-	<0.000	0.620 (0.451;0.789)
**Gender** *Reference – Men*	<0.000	0.548 (0.398;0.697)	<0.000	-0.393 (-0.580;-0.206)
**Self-rated health status**				
Reference – good				
average	0.022	0.302 (0.044;0.559)	0.363	-0.134 (-0.425;0.157)
poor	0.010	-0.350 (-0.613;-0.086)	0.596	0.081 (-0.220;0.382)
**Frailty Syndrome**				
Reference – non-frail				
pre-frail	0.894	0.013 (-0.182;0.208)	0.084	-0.188 (-0.401;0.026)
frail	0.727	-0.037 (-0.244;0.170)	0.002	0.356 (0.136;0.576)
**Hypertension**				
Reference – No	0.314	-0.071 (-0.212;0.069)	0.156	0.112 (-0.043;0.266)
**Diabetes**				
Reference – No	0.276	0.070 (-0.057;0.197)	0.622	-0.035 (-0.176;0.106)
**Thyroid diseases**				
Reference – No	0.441	0.053 (-0.082;0.187)	0.108	-0.121 (-0.269;0.027)
**Osteoporosis**				
Reference – No	0.825	-0.016 (-0.155;0.124)	0.764	0.024 (-0.131;0.178)
**Osteoarthritis**				
Reference – No	0.167	-0.090 (-0.219;0.038)	0.450	0.055 (-0.089;0.198)
**Hospitalization**				
Reference - Yes	0.887	-0.010 (-0.151;0.131)	0.089	-0.134 (-0.289;0.021)
**Alcohol drinking**				
Reference – Yes	0.199	-0.083 (-0.209;0.044)	0.077	0.126 (-0.014;0.266)
**Nutritional knowledge**				
Reference – good				
average	0.101	-0.121 (-0.265;0.024)	0.665	0.035 (-0.126;0.197)
lack	0.141	0.103 (-0.035;0.240)	0.373	-0.069 (-0.222;0.084)
**Dietary supplement use**				
Reference – Yes	0.870	0.011 (-0.124;0.147)	0.394	-0.065 (-0.215;0.085)

PRAL - potential renal acid load [mEq/day]; NEAP - net endogenous acid production [mEq/day]; CI - confidence interval
